# Health effects of identifying patients with undiagnosed obstructive sleep apnea in the preoperative clinic: a follow-up study

**DOI:** 10.1007/s12630-012-9694-8

**Published:** 2012-03-30

**Authors:** Vanita Mehta, Rajeev Subramanyam, Colin M. Shapiro, Frances Chung

**Affiliations:** 1 Department of Anesthesia, Toronto Western Hospital, University Health Network, University of Toronto, 399 Bathurst Street, Toronto, ON M5T 2S8 Canada; 2 Department of Psychiatry, Toronto Western Hospital, University Health Network, University of Toronto, Toronto, ON Canada

## Abstract

**Background:**

Undiagnosed obstructive sleep apnea (OSA) is a highly prevalent breathing disorder. The purpose of this study was to determine the effects of preoperative screening and subsequent treatment for OSA on the health of patients.

**Methods:**

We conducted a two-year follow-up study of patients previously enrolled in a large prospective study in which patients were given the STOP questionnaire for OSA screening (*n* = 2,467). All patients who underwent a polysomnography were considered eligible (*n* = 211) and were asked to complete a paper-based mailed questionnaire. The severity of OSA, comorbidities, and treatment modalities and their effects were evaluated from the returned questionnaire. Research ethics board approval was obtained and returning the questionnaire implied informed patient consent.

**Results:**

The response rate was 67%. One hundred twenty-eight (82%) of the 156 patients who responded had OSA established by polysomnography. Among these 128 patients with OSA, 88 (69%) were prescribed continuous positive airway pressure (CPAP) therapy and 40 (31%) were prescribed other (non-CPAP) treatment. Among those 88 patients receiving CPAP, 40 (45%) were compliant and 48 (55%) were non-compliant. The CPAP compliant patients had a greater reduction in medication for comorbidities than the CPAP non-compliant or the other treatment group (38% *vs* 3% *vs* 0%, respectively; *P* < 0.001). A significant improvement in snoring, sleep quality, and daytime sleepiness was reported by CPAP compliant users compared with CPAP non-compliant or other treatment groups (*P* < 0.001).

**Conclusion:**

The preoperative patients who were identified to have OSA and were compliant with CPAP use may have health benefits in terms of improved snoring, sleep quality, and daytime sleepiness. Timely diagnosis and treatment compliance may reduce symptoms of OSA and severity of associated comorbidities along with a reduction in medications.

## Résumé

**Contexte:**

Les apnées obstructives du sommeil non diagnostiqués (AOS) sont un trouble respiratoire hautement prévalent. L’objectif de cette étude était de déterminer les effets d’un dépistage préopératoire et du traitement subséquent de l’AOS sur la santé des patients.

**Méthodes:**

Nous avons mené pendant deux ans une étude de suivi de patients antérieurement inscrits dans une grande étude prospective au cours de laquelle le questionnaire STOP avait été administré pour le dépistage de l’AOS (*n* = 2 467). Tous les patients ayant bénéficié d’une polysomnographie ont été considérés admissibles (*n* = 211); il leur a été demandé de répondre à un questionnaire imprimé envoyé par courrier. La sévérité de l’AOS, les comorbidités et les modalités thérapeutiques ainsi que leurs effets ont été évalués à partir des questionnaires renvoyés. L’accord du comité d’éthique de la recherche a été obtenu et le retour du questionnaire impliquait le consentement éclairé du patient.

**Résultats:**

Le taux de réponse a été de 67 %. Cent vingt-huit patients sur les 156 ayant répondu (82 %) avaient une OSA mise en évidence par la polysomnographie. Parmi ces 128 patients souffrant d’AOS, 88 (69 %) ont reçu une prescription de traitement par pression positive continue des voies respiratoires (CPAP) et 40 (31 %) ont eu un autre traitement (non-CPAP). Parmi les 88 patients bénéficiant d’une CPAP, 40 (45 %) étaient observants et 48 (55 %) n’étaient pas observants. Les patients observants de la CPAP présentaient une plus grande baisse des traitements médicamenteux pour comorbidités par rapport aux patients non observants ou que les patients de l’autre groupe de traitement (respectivement, 38 %, 3 % et 0; *P* < 0,001). Une amélioration significative du ronflement, de la qualité du sommeil et de la somnolence diurne a été signalée par les utilisateurs de CPAP observants comparés aux utilisateurs de CPAP non observants ou aux patients des autres groupes de traitement (*P* < 0,001).

**Conclusion:**

Les patients qui avaient été identifiés en préopératoire comme ayant une AOS et qui suivaient le traitement par CPAP peuvent tirer des bénéfices pour la santé en termes de ronflements, de qualité du sommeil et de somnolence diurne. Le diagnostic en temps opportun et l’observance du traitement peuvent réduire les symptômes d’AOS, la sévérité des comorbidités associées et la prise de médicaments.

## Introduction

Obstructive sleep apnea (OSA) is a disorder characterized by intermittent closure of the upper airway during sleep, resulting in sleep fragmentation and night-time hypoxemia. Obstructive sleep apnea is the most prevalent breathing disorder with an incidence of one in four men and one in ten women.^1^ In the general population, moderately severe OSA is present in 11.4% of men and 4.7% of women.^2^,^3^ Obstructive sleep apnea is associated with several comorbidities, including acute myocardial infarction, heart failure, arrhythmias, refractory hypertension, cerebrovascular diseases, and metabolic syndrome.^4^-^9^

Anesthesiologists can play a crucial role in identifying patients with OSA. The screening of patients in the preoperative clinic provides an opportunity to identify patients at risk for OSA, thus directing them to the sleep physicians for better patient outcome. A number of questionnaire-based screening tools have been developed to screen OSA patients, and there have been two recent reviews on the topic of screening tests.^10^,^11^ The STOP questionnaire was developed and validated in our centre to screen surgical patients for OSA, and it has been shown to be effective in identifying patients at high risk for OSA.^12^,^13^

Among the different treatment modalities available, continuous positive airway pressure (CPAP) is the most efficient and the most widely used. Continuous positive airway pressure is effective in reducing the nocturnal events of OSA, and it may provide subjective benefits, such as improvement in daytime sleepiness, cognitive function, and well-being.^14^,^15^ Continuous positive airway pressure has also been shown to decrease blood pressure in OSA patients with hypertension, and it improves glucose control in diabetic patients with severe OSA.^16^,^17^ However, while CPAP is highly effective in controlling symptoms of sleep apnea, the device is cumbersome, and data show only moderately satisfactory compliance.^18^,^19^

The main objectives of the study were to determine the health benefits of diagnosing OSA in the surgical patients in terms of improved snoring, sleep quality, daytime sleepiness, subjective fatigue, and reduction of medication to treat coexisting medical diseases.

## Methods

### Study design and patient population

This study was a two-year follow-up study of the patients enrolled in a previous large prospective study designed to develop a screening tool for OSA (STOP questionnaire). Patients who had submitted to a polysomnography (PSG) test were considered eligible and were asked to complete a questionnaire related to their health condition.

Polysomnography is the “gold standard” diagnostic tool for assessing sleep-disordered breathing. It requires an overnight stay in a sleep laboratory with monitoring of oxygen saturation, heart rate, sleep stage (via electroencephalography), jaw muscle tone (via electromyography), air flow, nasal flow, and chest and abdominal movement. Respiratory parameters are measured to determine apneas and hypopneas. Apnea has been defined as a complete cessation of airflow lasting ≥ ten seconds, and hypopnea has been defined as a ≥ 50% reduction in respiratory airflow lasting longer than ten seconds and associated with an arousal or oxygen desaturation by ≥ 4%. The apnea-hypopnea index (AHI), i.e., the number of obstructive events per hour, is the measurement most commonly used to quantify OSA: mild = 5-15; moderate = greater than 15-30; severe = greater than 30.^20^

Among the patients enrolled in the original study (*n* = 2,467), 416 consented to an overnight PSG, and 211 of those underwent the test due to “no shows”. These 211 patients underwent various surgeries (neurosurgical, orthopedic, spine, urologic, obstetric, general surgical (except bariatric), and otorhinolaryngologic surgeries) at Toronto Western and Mount Sinai Hospitals in Toronto. The patients were then referred to sleep physicians at the Sleep Clinic of Toronto Western Hospital within three months of surgery. The sleep physicians subsequently prescribed the different treatments depending on the diagnosis and followed up with the patients for treatment compliance. In the present study, we conducted a follow-up study of these 211 patients at two years.

### Questionnaire development

In order to contact these patients and obtain information, we devised a new questionnaire/template (for this study) to administer to patients (Appendix). The questionnaire was designed in three sections. The first and second sections of the questionnaire were derived from the standard questions that were administered routinely to patients in our sleep clinic on their first and follow-up visits. The first section included a set of questions on diagnosis, treatment prescription, and changes in OSA symptoms (e.g., snoring), whereas the second section had questions on comorbidities. The third section of the questionnaire included two validated sets of questions, i.e., the Epworth Sleepiness Scale and the Fatigue Severity Scale.

The questions on CPAP compliance (first section of questionnaire) were focused on details regarding the number of nights per week and hours per night of CPAP use. Self-reported CPAP compliance was defined as the use of CPAP for > four hours per day for > five nights per week.^21^ Patients were asked to report any preexisting medical conditions, such as hypertension, congestive heart failure, arrhythmias, diabetes mellitus, asthma, gastroesophageal reflux disease, and/or depression. Patients were also asked to specify any change or reduction in prescribed medications, such as a reduction in antihypertensive drugs, antiarrhythmic drugs, hypoglycemic drugs, antidepressant drugs, and antacids. The reduction in medications was defined as a reduction in either the dosage or the total number of prescribed medications.

The Fatigue Severity Scale measures the fatigue level experienced by the patients.^22^ Patients were asked questions to respond to nine different statements on a scale of 1-7 (1 = strongly disagree, and 7 = strongly agree). The final score is the mean of the nine items, and a higher score indicates a higher fatigue level. The Fatigue Severity Scale was measured before and two years after the treatment prescription.

The Epworth Sleepiness Scale measures the general level of daytime sleepiness as experienced by the patients.^23^ Patients were asked to rate their chance to fall asleep during different routine daytime situations on a scale of 0-3 (0 = little or no chance of dozing, and 3 = a high chance of dozing. The final score is the total score of eight items and ranges from 0 to 24. A score ≥ 10 is considered abnormal, and a score > 14 may suggest a more significant degree of daytime hypersomnolence. The Epworth Sleepiness Scale was measured at two years after treatment prescription.

### Questionnaire dissemination

The questionnaires specifically designed for the study patients were mailed to all 211 patients with an accompanying cover letter. The cover letter provided information about voluntary participation with no financial remuneration and instructions to patients not to identify themselves on the questionnaire. The questionnaires were numbered, however, to identify the respondents. The patients were instructed to return the completed questionnaire in the pre-stamped envelopes provided. If there was no response at two weeks, a phone call was made to ensure completion of the mailing process and to encourage the patients to return the questionnaire with their responses. At four weeks, non-respondents were requested to reply to the questionnaire during a phone call. The returned questionnaires and sleep study charts of the participants were reviewed by one of the investigators (V.M.).

The study protocol was approved by the Hospital Research Ethics Board (University Health Network, Toronto, ON, Canada) (May 2010, # 10-0067-AE). The requirement of a separate consent form was waived by the Research Ethics Board, since returning the questionnaire implied informed consent of the participant.

### Statistics

The statistical analysis was performed using SPSS^®^ v17 (SPSS INC., Chicago, IL, USA) and MedCalc software (MedCalc software, Mariakerke, Belgium). The data were analyzed using descriptive statistics. The normality of data distribution was tested using the D’Agostino-Pearson test. Data are represented with number of patients, mean (SD) or mean (95% confidence interval of mean) as appropriate. Continuous variables, such as age, body mass index (BMI), neck circumference, Epworth Sleepiness Scale, and Fatigue Severity Scale were analyzed using the Student’s *t* test. Use of AHI and CPAP were analyzed with the nonparametric Mann-Whitney U test. Nominal variables, such as sex and comorbidities were analyzed using the Chi square test and Fischer’s exact test where appropriate. In all cases, *P* < 0.05 was considered statistically significant.

## Results

We mailed the questionnaires during June and July 2010. One hundred fifty-six of the 211 patients who underwent PSG and were eligible for the study were ultimately included in the final analysis (Figure). Fifty-five patients were excluded for various reasons: 24 patients could not be contacted due to change of address and/or phone number, 28 patients declined to participate, and three patients were deceased. Questionnaires were returned by 141 patients, and 15 patients completed the questionnaire over the phone.

**Figure:**
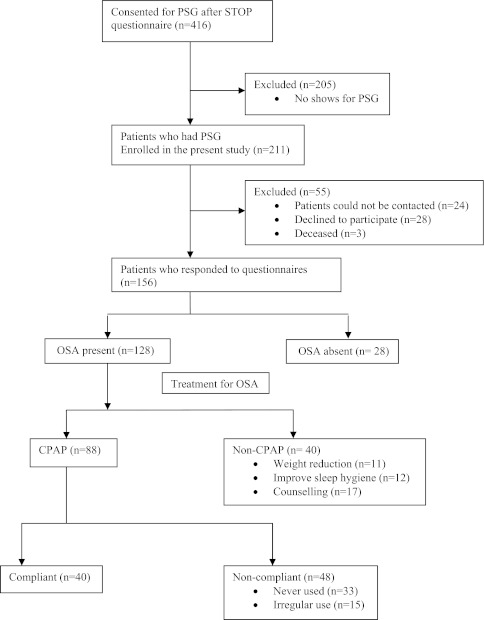
Patient flow chart

As confirmed by PSG two years prior to our contact, 128 (82%) of the 156 patients who responded to the questionnaires had a diagnosis of OSA while 28 (18%) had no diagnosis of OSA. The demographic data of the participants are shown in Table 1. The OSA patients were older with a higher BMI and a larger neck circumference than the non-OSA patients. The AHI was also higher in OSA patients *vs* non-OSA patients [20.9 (21.6) *vs* 1.8 (1.6), respectively].

**Table 1:** Demographic data

	OSA (*n* = 128)	No OSA (*n* = 28)
Sex (male/female)	72/56	9/19
Age (yr)	61 (12)	51 (15)
BMI (kg·m^−2^)	31.1 (7.0)	26.8 (5.4)
Neck circumference (cm)	39.2 (3.6)	34.8 (4.2)
Pre-existing conditions *n* (%)		
Hypertension	64 (50)	11 (39.3)
CVD	10 (7.8)	4(14.3)
Diabetes	22 (17.2)	1 (3.6)
Asthma	11 (7.1)	2 (8.6)
GERD	22 (17.2)	6 (21.4)
Depression	31 (19.9)	5 (17.9)
AHI	20.9 (21.6)	1.8 (1.6)

Data are expressed as mean (standard deviation) or numbers (%). OSA = obstructive sleep apnea; BMI = body mass index; CVD = cardiovascular diseases, including angina, arrhythmia, and/or congestive cardiac failure; GERD = gastroesophageal reflux disease; AHI = apnea-hypopnea index

Among 128 patients with OSA, 88 (69%) were prescribed CPAP therapy and 40 (31%) were prescribed non-CPAP treatment modalities: advice on weight loss (*n* = 11); improve sleep hygiene (*n* = 12); counselling for follow-up and repeat PSG (*n* = 17). According to the patients’ responses in the questionnaire regarding CPAP compliance, 40 (45%) were compliant and 48 (55%) were non-compliant with the prescribed CPAP treatment. The compliant group used the CPAP on an average of six days per week with a continuous use for more than six hours per night. The difference in CPAP compliance between the CPAP compliant and the CPAP non-compliant groups was statistically significant (Table 2). The patients receiving non-CPAP treatment were younger, had a low BMI, and a substantially lower AHI compared with the CPAP users.

**Table 2:** Data on patients recommended with the different treatments

	CPAP users		Non-CPAP treatment (*n* = 40)
	Compliant (*n* = 40)	Non-compliant (*n* = 48)	
Sex (male/female)	20/20	29/19	23/17
Age (yr)	63.2 (12)	63.7 (10)	54.9 (12)
BMI (kg·m^−2^)	33.2 (9)	30.5 (6)	29.7 (6)
Neck circumference (cm)	39.9 (5)	39.2 (4)	38.5 (4)
AHI	31.8 (28)	23.6 (18)	6.7 (4)
CPAP use			
days/week	6.5 (0.9)*	0.5 (0.9)	N/A
hours/night	6.3 (1.2)*	0.7 (1.1)	N/A

Data expressed as number of patients or mean (standard deviation). Other treatment = weight reduction and improving sleep hygiene. *P < 0.001 (CPAP compliant *vs* CPAP non-compliant). CPAP = continuous positive airway pressure; BMI = body mass index; AHI = apnea-hypopnea index; N/A = not applicable

### Effects of treatment on comorbidities

Thirty-eight percent of the CPAP compliant patients reported a reduction in medication *vs* 3% of the CPAP non-compliant patients (*P* < 0.001) (Table 3). Among the 38% of patients with a reduction in medication, 15% of patients in the compliant group had a reduction in the total number of prescribed medications. In the CPAP non-compliant group, no patients had a reduction in the total number of medications. Also, the patients receiving non-CPAP treatment did not report any reduction in medication for their coexisting medical conditions.

**Table 3:** Percentages of patients reporting reduction in medication after obstructive sleep apnea treatment

Comorbidities	CPAP users		Non-CPAP treatment (*n* = 40) (%)	P* value
	Compliant (*n* = 40) (%)	Non-compliant (*n* = 48) (%)		
Hypertension	28	5	0	0.05
Diabetes	12	0	0	1
CVD	12	0	0	1
Asthma	0	0	0	N/A
GERD	36	0	0	N/A
Depression	14	0	0	0.43
Overall medication reduction	38	3	0	< 0.001

*Represents *P* value of the test difference between the CPAP compliant and non-compliant groups. CPAP = continuous positive airway pressure; CVD = cardiovascular diseases, including angina, arrhythmia, and /or congestive cardiac failure; GERD = gastroesophageal reflux disease; N/A = not applicable

### Effects of treatment on OSA symptoms

Improvement in snoring and sleep quality occurred in 36 (90%) and 35 (87%) of the CPAP compliant patients, respectively, *vs* four (8%) of the CPAP non-compliant patients (*P* < 0.001). Improvement in tiredness was reported in 33 (82%) of CPAP compliant patients *vs* four (8%) patients who were non-compliant and one (3%) patient who received non-CPAP treatment modality (Table 4). The major side effects of CPAP use reported by patients were dry mouth (25%), dry nose (15%), and mask discomfort (15%). Other complaints included occasional morning rhinorrhea, choking sensation, and “mask falling off”.

**Table 4:** Outcome measures in patients with obstructive sleep apnea

Outcome	CPAP users		Non-CPAP treatment (*n* = 40)
	Compliant (*n* = 40)	Non-compliant (*n* = 48)	
Improvement in snoring *n* (%)	36 (90)*	4 (8)	N/A
Improvement in sleep quality *n* (%)	35 (87)*	4 (8)	N/A
Improvement in tiredness *n* (%)	33 (82)*^†^	4 (8)	1 (3)
Weight loss *n* (%)	10 (25)	8 (17)	7 (18)
Physician visits	4.0 (3.1) (3.0 to 5.0)	4.0 (3.7) (3.0 to 5.1)	3.5 (2.6) (2.7 to 4.4)
Epworth Sleepiness Scale	5.3 (2.6) (4.4 to 6.1)*^‡^	8.6 (5.1) (7.1 to 10)	7.4 (4.2) (6.1 to 8.7)
Fatigue Severity Scale			
Pre-treatment	4.2 (1.4) (3.7 to 4.6)^§^	3.3 (1.7) (2.8 to 3.8)	3.2 (1.5) (2.7 to 3.7)
Post-treatment	2.7 (1.5) (2.2 to 3.2)	2.8 (1.6) (2.4 to 3.3)	2.7 (1.8) (2.1 to 3.2)

Data expressed as number (percent) or mean (standard deviation) (95% confidence interval for mean) *P < 0.001(CPAP compliant *vs* CPAP non-compliant groups). ^†‡^ *P* < 0.001, *P* < 0.007 (CPAP compliant *vs* Non-CPAP treatment groups). ^§^ *P* < 0.001 (pre-treatment *vs* post-treatment in CPAP compliant group). CPAP = continuous positive airway pressure; N/A = not applicable

### Epworth Sleepiness Scale & Fatigue Severity Scale

The results of the Epworth Sleepiness Scale in the CPAP compliant patients were significantly lower than those in the CPAP non-compliant patients (Table 4) and lower than the results in patients receiving non-CPAP treatment. The results of the Fatigue Severity Scale in the CPAP compliant patients showed a significant improvement in their subjective feeling of fatigue post-treatment *vs* pre-treatment, but there was no difference between CPAP compliant and CPAP non-compliant patients (Table 4). The patients with non-CPAP treatment modalities had improved Fatigue Severity Scale results post-treatment *vs* pre-treatment, however the difference was not statistically significant.

## Discussion

This study showed that > 80% of CPAP compliant patients showed improved snoring, sleep quality, daytime sleepiness, and fatigue, respectively. Thirty-eight percent of CPAP compliant patients had self-reported reduction in medication for coexisting diseases. The administration of the STOP questionnaire as a screening tool for OSA was associated with health benefits for patients who were referred to the sleep clinic and consequently experienced a reduction in the severity of OSA-associated comorbid conditions.

We chose to follow the surgical patients screened and diagnosed with OSA with a mailed questionnaire and a follow-up phone call. We received a good response rate of 67% with this follow-up. This method was used successfully in another study regarding long-term follow-up of OSA.^24^

Compliance plays an important role in observing the effects of the treatment. In two prospective studies evaluating the factors of CPAP adherence and the rate of long-term compliance, the rate of CPAP compliance ranged from 28-84%.^25^,^26^ In our study, the rate of CPAP compliance was 45% at two years. Compared with the CPAP non-compliant patients, the compliant patients had a higher AHI at the start of this study with more severe symptoms of obstructive sleep apnea. Their condition showed marked improvement with CPAP use, a major reason for CPAP compliance. On the other hand, patients with a non-CPAP treatment prescription reported only minimum benefits from treatment, most likely due to mild OSA symptoms.

Non-compliance with CPAP has been a major issue in treating the OSA patients with CPAP, and it can have a significant impact on the mortality associated with untreated OSA. Marin *et al.* have shown that the patients who are untreated or non-compliant with OSA treatment tend to have high mortality.^27^ Another study by Marshall *et al.* showed that the hazard ratio of early death is 4.4-6.2 for untreated moderate to severe sleep apnea after controlling for different covariates such as age, sex, BMI, smoking status, and total cholesterol level.^28^ The rate of CPAP non-compliance in our study was 55%, and this non-compliance to treatment partly explains the lack of benefit reported by our CPAP non-compliant group. Our result is also consistent with other reports that 50% of patients abandoned the use of CPAP within one year of therapy.^29^-^32^ The major reasons for CPAP non-compliance were mild symptoms, cumbersome device, and side effects like dry mouth, dry nose, and mask discomfort.

A strong association between OSA and hypertension has been established by a number of studies.^33^,^34^ A prospective study identified that even minimally elevated AHI at baseline was associated with 42% (95% confidence interval, 13 to 78) increased odds of developing hypertension over a four-year follow-up period.^35^ The effectiveness of reducing blood pressure by treating OSA with CPAP has been shown by several intervention studies.^36^-^39^ In our study, a larger percentage of CPAP compliant patients self-reported a reduction in the dosage of anti-hypertensive medication *vs* other groups of patients. Most importantly, we found that the percentage of patients with a reduction of medication for comorbidities was significantly higher in patients compliant to CPAP *vs* other groups of patients.

Excessive daytime sleepiness is a cardinal feature of the OSA. A significant, progressive increase in Epworth Sleepiness Scale score was shown to be associated with a severity of OSA.^40^ Studies, including placebo or sham-CPAP controlled studies, have shown an improvement in daytime sleepiness after treatment of OSA.^41^,^42^ In context with these studies, our patients who were treated and compliant with CPAP reported an improvement in their daytime sleepiness compared with other treatment groups.

Fatigue often coexists in patients with OSA as a consequence of sleep fragmentation. In a recent prospective observational study, subjective fatigue was identified as an independent manifestation of sleep disorder along with subjective sleepiness.^43^ Before initiation of the treatment, our patients with severe OSA symptoms reported higher fatigue levels, which subsequently improved following CPAP compliance.

From the economical perspective, OSA imposes significant health care expenditures on finite resources due to its association with various comorbid diseases, particularly hypertension, ischemic heart disease, and diabetes. Many studies evaluating the economic implications of sleep disorders have shown that the health care expenditures for OSA decline significantly once the illness is properly treated with weight reduction and/or CPAP.^44^,^45^ Our study has shown that surgical patients compliant with CPAP use benefited from OSA diagnosis and treatment with significant improvement in daytime sleepiness and fatigue. The patients with non-CPAP treatment recommendations also reported some improvement in their daytime somnolence and fatigue levels; however, the results were not statistically significant. The CPAP compliant patients in our study had medication reduction for comorbidities, which might reduce health care expenditures as indicated by studies analyzing the economic burden of OSA diagnosis and treatment.^46^,^47^

There are certain limitations of the study. The results of the study are based on a small sample size and sub-group analysis of a large cohort study, which is a major limitation. Also, the study is not a randomized clinical trial to determine the benefits of CPAP and other modalities of treatment, but it is able to provide further evidence for the beneficial effects of CPAP by comparison of CPAP compliant patients with a natural control group, the CPAP non-compliant patients. However, it is difficult to determine if the beneficial effects attributed to CPAP therapy were a result of a selection bias. Also, the initial parts of questionnaires are non-validated and categorized as binary, as opposed to a scale. We chose to use binary as we use this approach routinely at our sleep clinics. Another possible limitation is the risk of recall bias since patients had to rely on their memory to answer the questionnaire. The self-reported CPAP compliance may be significantly higher than that determined by the record stored in the CPAP machine. Also, preoperative PSG testing, justified on the basis of a STOP score suggestive of OSA, may not always be possible before surgery depending on how soon the patient is evaluated before the actual surgery. However, there are still health benefits from less daytime sleepiness, less fatigue, and reduction of medications, even though the referrals to a sleep physician may occur after surgery. Although our study did not analyze the economic burden on OSA patients, it does provide evidence of a possible decline in health care expenditures based on the health benefits perceived by the patients.

In conclusion, this study suggests that those preoperative patients who were identified as having OSA and who were compliant with CPAP use may have health benefits in terms of improved snoring, better quality of sleep, and lower levels of daytime sleepiness and fatigue. Timely diagnosis and treatment compliance may reduce symptoms of OSA and the severity of associated comorbidities with a reduction in medication. Since the findings are based on a small subgroup of CPAP compliant patients, a larger study evaluating the clinically significant outcomes needs to be performed to further strengthen our suggested hypothesis.

## Appendix A follow-up questionnaire for screening of obstructive sleep apnea

We would be grateful if you would answer the following questions, which should take about 10 to 15 minutes to complete. As mentioned in the information letter, we are now trying to determine whether the diagnosis of sleep apnea, made known by the screening in the preoperative clinic, and its timely management did indeed provide beneficial effects. Furthermore, we want to determine whether you had any change in symptoms, for example, snoring, daytime sleepiness, fatigue, and tiredness. We also would like to know how well you tolerate CPAP therapy if it was prescribed for you, and whether you have any long-term benefits after diagnosis and treatment, such as a reduction in blood pressure medications, fewer symptoms of congestive heart failure, and better control of diabetes mellitus, if you previously had any of those conditions.

We appreciate your time and participation in this study. Your participation in this study is entirely voluntary. You can choose not to answer any of the questions or you may skip some questions if you wish.

Please answer the following questions to the best of your knowledge:
